# Dysfunction of circulating CD3^+^CD56^+^ NKT-like cells in type 2 diabetes mellitus

**DOI:** 10.7150/ijms.83317

**Published:** 2023-04-02

**Authors:** Ling Tang, Hui Wang, Kangli Cao, Cairui Xu, Along Ma, Meijuan Zheng, Yuanhong Xu, Min Zhang

**Affiliations:** Department of Clinical Laboratory, First Affiliated Hospital of Anhui Medical University, Hefei, Anhui, China

**Keywords:** type 2 diabetes mellitus, NKT-like cells, Tim-3, dysfunction

## Abstract

Type 2 diabetes mellitus (T2DM) is associated with increased incidence and mortality of many cancers and infectious diseases. CD3^+^CD56^+^ NKT-like cells play pivotal roles in tumor surveillance and infection control. However, little is known about potential alterations in circulating NKT-like cells in T2DM patients. In this study, we found that the frequency and absolute counts of circulating NKT-like cells were significantly lower in patients with T2DM compared to healthy volunteers. Moreover, in T2DM patients, NKT-like cells were impaired in their production of IFN-γ and TNF-α as well as degranulation capacity. The expression of activating receptor NKG2D was markedly decreased on NKT-like cells in T2DM patients, while the expression of inhibitory receptors Tim-3 and LAG-3 was upregulated. In detail, Tim-3^+^NKT-like cells expressed higher LAG-3 and less IFN-γ and TNF-α compared to Tim-3^-^NKT-like cells. Importantly, we further found that the expression of Tim-3 in NKT-like cells from T2DM patients correlated positively with glycated hemoglobin (HbA1c) and fasting blood glucose (FBG) levels, as well as with diabetes duration. In conclusion, these results indicate that NKT-like cells from T2DM patients display an exhausted phenotype and reduced functionality. Moreover, Tim-3 expression on NKT-like cells likely serves a novel biomarker for duration of T2DM.

## Introduction

Type 2 diabetes mellitus (T2DM) is a worldwide disease associated with low-grade inflammation, impaired immunity, insulin resistance, and hyperglycemia 1-3. A bidirectional link between metabolic and immune activities exists in T2DM; inflammation plays an essential role in the promotion of metabolic abnormalities (such as hyperglycemia and obesity), which regulate in turn immune cell functions 3. Immune system dysfunction, reflected by homeostatic dysregulation of T cell populations, dysfunction of NK and B cells, as well as abnormal polarization of macrophages, provides a reasonable explanation for the higher susceptibility to infections in patients suffering from T2DM 2.

CD3^+^CD56^+^ NKT-like cells share both receptor expression profiles and functional characteristics with NK cells and T cells and possess dual, innate and adaptive, immune functions. NKT-like cells are a heterogeneous population which comprises TCRγδ T cells and mucosal-associated invariant T (MAIT) cells, as well as CD4^+^ cells and CD8^+^ cells 4. These populations can be activated by TCR signals and non-MHC-restricted pathways 5, 6. A hallmark of NKT-like cells is the capacity to rapidly elicit their effector functions, including cytokine secretion and cytotoxicity 7. NKT-like cells have been revealed to be involved in cancers 8, 9, autoimmune diseases 10, 11 and infections 12, 13. In hepatocellular carcinoma patients, circulating NKT-like cells are functionally exhausted and are characterized by downregulation of NKG2D, upregulation of programmed cell death protein 1 (PD-1), T-cell immunoglobulin and mucin structural domain protein 3 (Tim-3), and cytotoxic T-lymphocyte antigen 4 (CTLA-4), and impaired functionality 9. However, the influence of T2DM on NKT-like cells remains controversial. According to the few available studies on this topic, circulating NKT-like cells counts are significantly higher in pre-diabetes and remain unchanged in new-onset T2DM patients, compared to healthy controls 14, 15.

Tim-3 is an immune inhibitory receptor expressed on NK cells, T cells, and mast cells 16. It was suggested that inhibition of the Galectin-9/Tim-3 pathway might be a new approach for the treatment of type 1 diabetes 17. We recently reported that in T2DM patients, increased expression of Tim-3 is associated with NK cell dysfunction and apoptosis 18. However, it remains unclear whether T2DM also affects Tim-3 expression and function in NKT-like cells.

To investigate the phenotypic and functional characteristics of circulating NKT-like cells in patients with T2DM, we evaluated their absolute number and frequency, susceptibility to apoptosis, and expression of functional markers, and performed a detailed analysis of the expression of inhibitory and activating receptors. Our results suggest that phenotypic alterations and dysfunction of NKT-like cells occur in T2DM patients. Moreover, Tim-3 expression on NKT-like cells likely serves a novel biomarker for duration of T2DM.

## Materials and Methods

### Study subjects

The study included 54 T2DM and 33 age- and sex-matched healthy controls (HC). The clinical characteristics of the patients and controls are shown in **Table 1**. T2DM patient inclusion criterion was more than 2 years since the diagnosis of T2DM. Exclusion criteria included stage 3 and 4 chronic kidney disease, history of malignancy, acute diseases, immunosuppressive therapy, chronic or acute hepatitis B, or AIDS. In addition, female and male subjects aged under 40 or over 80 years were excluded. The diagnostic criteria for T2DM are based on the World Health Organization (WHO) definitions 19. The study was approved by the local Ethics Committee of The First Affiliated Hospital of Anhui Medical University.

### Isolation of peripheral blood mononuclear cells

Peripheral blood mononuclear cells (PBMCs) were isolated from freshly obtained peripheral blood from T2DM patients and HCs using Ficoll-Paque (TBD Science, #LTS1077) gradient centrifugation. Briefly, fresh blood was diluted with 1×phosphate buffered saline (1×PBS), gently overlaid on Ficoll, and centrifuged at 380 g for 20 min. After centrifugation, the middle layer was harvested and washed with 1×PBS.

### Antibodies and flow cytometry analysis of immune cell phenotype

Flow cytometry staining of PBMCs was conducted using the following anti-human monoclonal antibodies (obtained from BioLegend): CD3 (HIT3a, PerCP-Cy5.5), CD56 (HCD56, BV510), CD45 (2D1, APC-Cy7), Tim-3 (F38-2E2, PE), NKG2A (S19004C, APC), TIGIT (A15153G, APC), NKG2D (1D11, APC), NKP46 (9E2, FITC), CD27 (M-T271, FITC), CD69 (FN50, PE-Cy7), LAG-3 (11C3C65, BV421), TCR Vα24-Jα18 (6B11, PE), TNF-α (W19063E, PE), IFN-γ (4S.B3, APC), and CD107a (H4A3, PE-Cy7). An isotype control immunoglobulin G was used as a control. Anti-PD-1 (EH12.1, BV421) and anti-Ki67 (B56, Alexa-Fluor 647) antibodies were purchased from BD Bioscience. PBMCs were incubated with antibodies at 4℃ for 30 min. Dead cell exclusion was not performed in this experiment. Cell staining was evaluated using a BD FACSCanto flow cytometer, and data analysis was performed with FlowJo software v10 (TreeStar, USA).

### Flow cytometric analysis of immune cell function

For intracellular cytokine assays, isolated PBMCs were stimulated with RPMI-1640 (HyClone) containing 10% fetal bovine serum (FBS, Biosharp, China) and 50 ng/ml phorbol 12-myristate 13-acetate, 1 µg/ml ionomycin, and 10 µg/ml monensin (all from Sigma Aldrich) for 4 h. CD107a antibody was added at the start of the incubation. PBMCs were then stained with the membrane markers. The cells were then fixed, permeabilized, and labeled with specific cytokine-targeting antibodies. Dead cell exclusion was not performed in this experiment. After washing off excess antibodies, signal detection was carried out by flow cytometry.

### Apoptosis detection

Apoptosis detection in NKT-like cells was performed using an APC Annexin V Apoptosis Detection Kit (BioLegend). Cells were washed twice with PBS and resuspended in 1× binding buffer at a concentration of 1×10^6^ cells/ml. Then, a 100 µL sample (1×10^5^ cells) was transferred to a 5 ml test tube with 5 µL APC Annexin V and 7-AAD. The cells were then gently vortexed and incubated for 15 min in the dark at room temperature (25℃). Finally, 400 µL of 1× binding buffer was added to each tube before analysis by flow cytometry.

### High glucose culture system of PBMC

Freshly isolated healthy controls PBMCs were cultured in glucose-free RPMI-1640 medium with 10% FBS containing 5.5 mM, 25 mM and 50 mM glucose for 24h, 48h and 72h. Then, the expression of Tim-3 on NKT-like cells was analyzed.

### Statistical analyses

Statistical analysis was performed using GraphPad Prism 5.0. All quantitative data were expressed as mean ± standard deviation (SD). Comparisons between two groups were made using an unpaired t-test or Mann-Whitney U test, depending on whether they conformed to a normal distribution. Pearson's correlation test was used for correlation analysis. *P* < 0.05 was considered significant.

## Results

### Circulating CD3^+^CD56^+^ NKT-like cell numbers are reduced in T2DM patients

**Figure 1:**
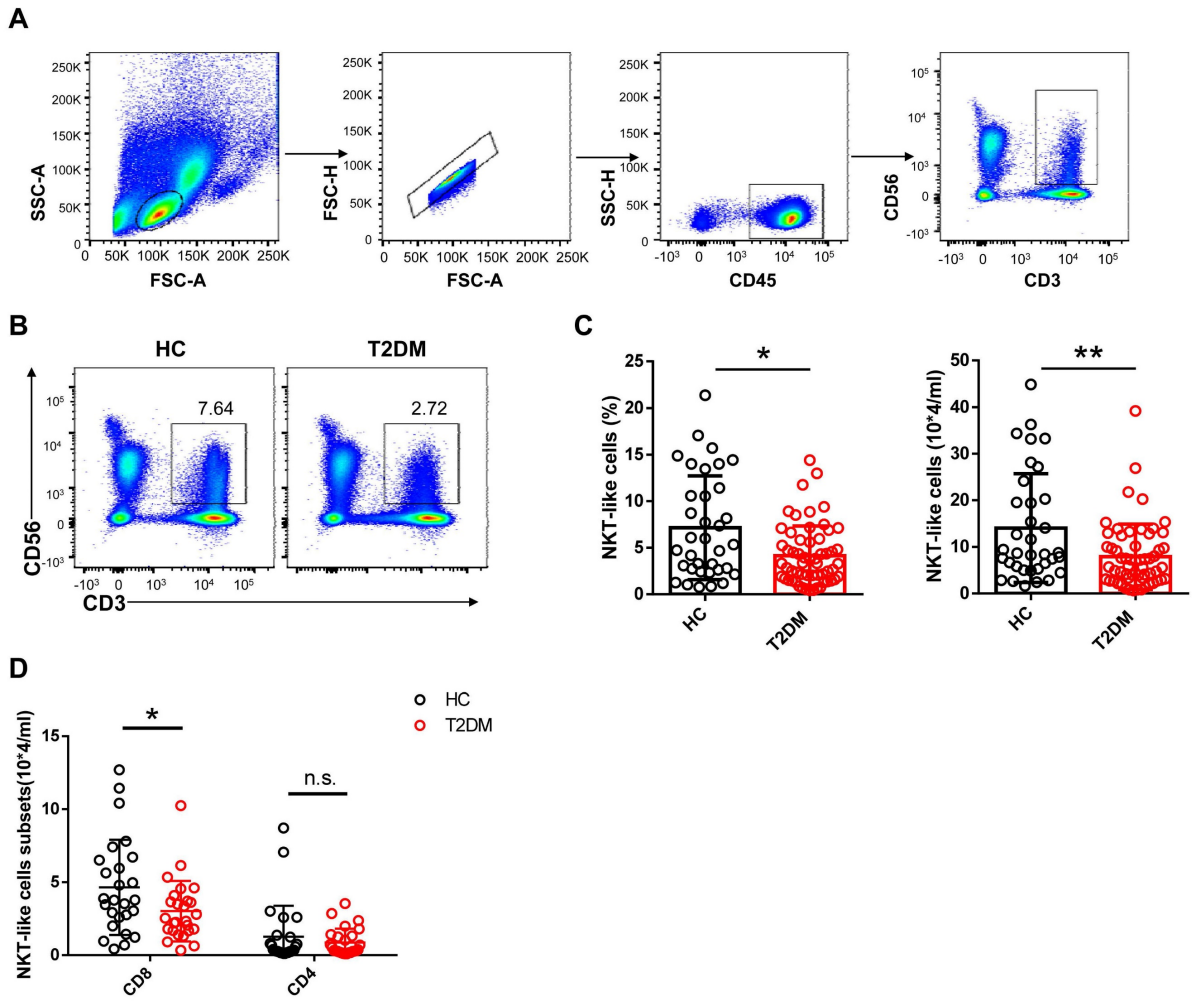
Number and frequency of circulating CD3^+^CD56^+^ NKT-like cells are decreased in T2DM patients. (**A**) Sequential strategy for gating NKT-like cells from PBMCs. (**B**) Representative flow cytometry plots of CD3^+^CD56^+^ NKT-like cells in T2DM patients and healthy controls (HC). (**C**) Flow cytometry estimations of frequency and absolute number of NKT-like cells in T2DM patients and HC. (**D**) Flow cytometry estimations of CD4^+^ and CD8^+^ NKT-like cell subsets in T2DM patients and HC. In (B) and (C), each dot represents a different individual and results are presented as the mean ± SEM; *P<0.05, **P<0.01, n.s., not significant.

**Figure 2:**
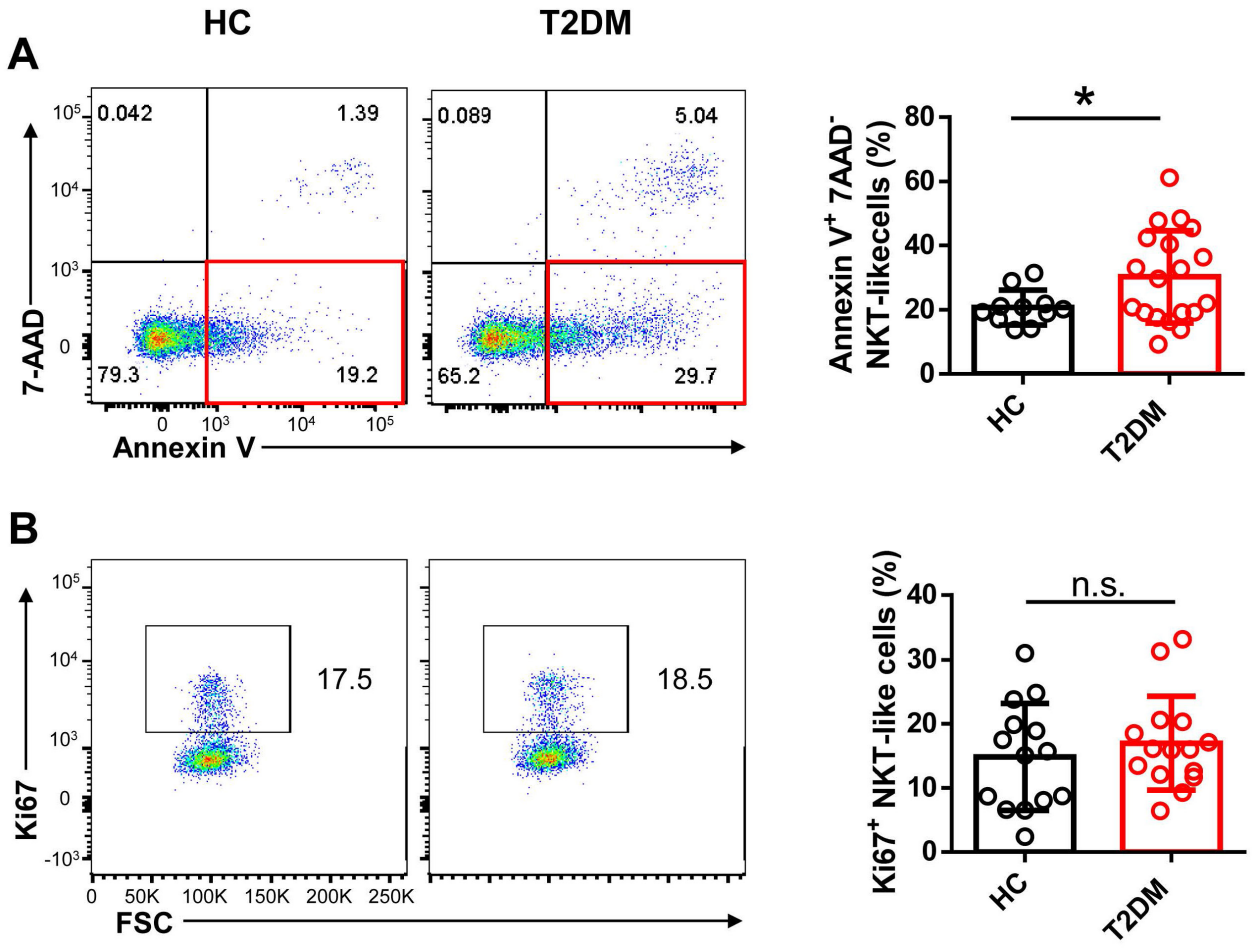
Peripheral blood NKT-like cells from patients with T2DM are prone to apoptosis. Comparison of early apoptosis via Annexin V^+^ 7-AAD^-^ staining (**A**) and proliferation status via Ki67^+^ labeling (**B**) in NKT-like cells from T2DM patients and healthy controls (HC). Data are presented as the mean ± SEM. *P<0.05, n.s., not significant.

To evaluate potential alterations in CD3^+^CD56^+^ NKT-like cell abundance and function in T2DM patents, we recruited 54 patients who have been diagnosed as T2DM for about 10 years and 33 age- and sex-matched healthy controls (HC) and analyzed the frequency and absolute count of NKT-like cells in peripheral blood (**Table 1**). We found that both the frequency and absolute number of NKT-like cells were much lower in patients with T2DM than in HC (**Figure 1A-C**). CD8^+^ cells were the largest subset of NKT-like cells, and their absolute number was also significantly decreased in T2DM patients (**Figure 1D**). The frequency of TCR Vα24-Jα18^+^ NKT cells was comparable between T2DM patients and healthy controls (**Supplementary Figure S1A, B**). To explore the mechanism mediating this reduction, we evaluated early apoptosis (via Annexin V^+^7-AAD^-^ staining) and proliferation status (via Ki67 labeling) in NKT-like cells from T2DM patients and HC. Results showed that the frequency of apoptotic NKT-like cells was higher in T2DM patients than in HC (**Figure 2A**), while the frequency of proliferative (Ki67^+^) NKT-like cells was similar (**Figure 2B**). These results suggest that NKT-like cells from T2DM patients are more prone to apoptosis, which would explain their lower circulating levels.

### NKT-like cells from T2DM patients exhibit an exhausted phenotype

**Figure 3:**
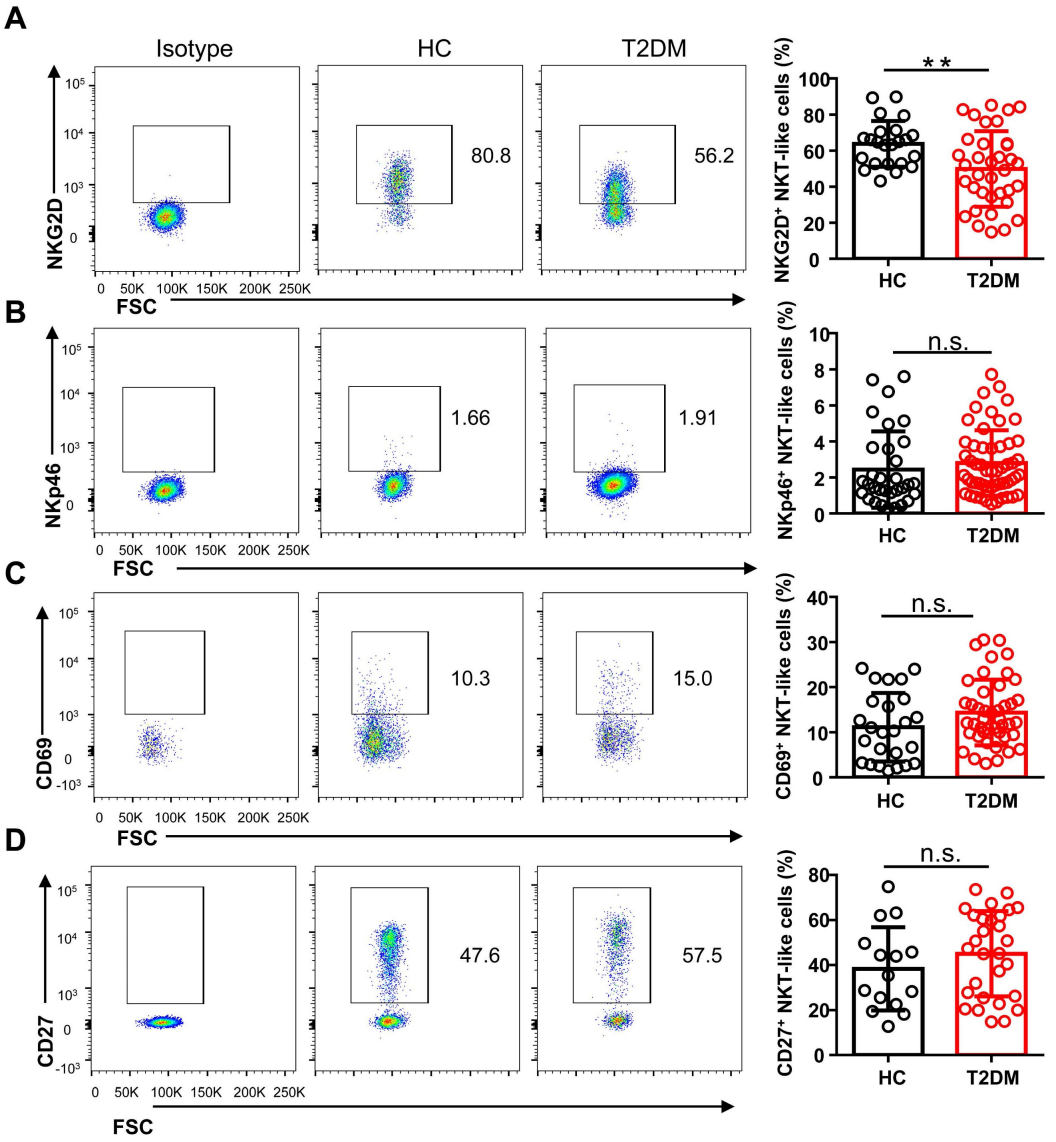
NKT-like cells from T2DM patients display lower levels of activating receptors. Representative flow cytometry plots and proportions of NKG2D^+^ (**A**), NKp46^+^ (**B**), CD69^+^ (**C**), and CD27^+^ (**D**) NKT-like cells in patients with T2DM and healthy controls (HC). In the quantification histograms, each dot represents a different individual and results are presented as the mean ± SEM; **P<0.01, n.s., not significant.

**Figure 4:**
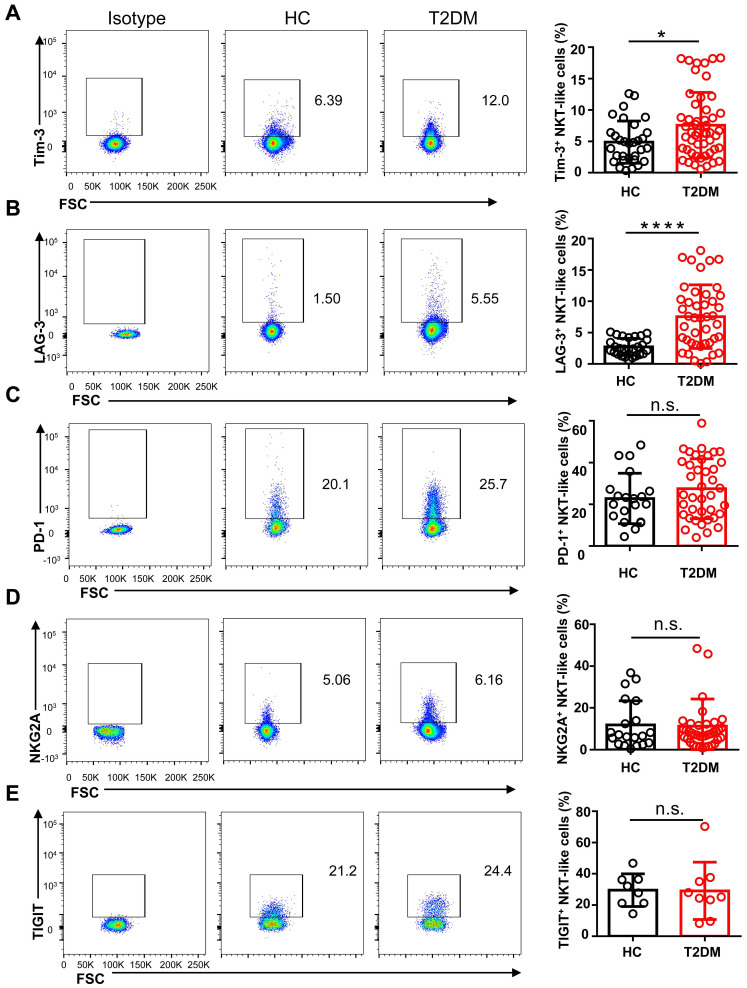
NKT-like cells from T2DM patients express higher levels of inhibitory receptors. Representative flow cytometry plots and proportions of Tim-3^+^ (**A**), LAG-3^+^ (**B**), PD-1^+^ (**C**), NKG2A^+^ (**D**), and TIGIT^+^ (**E**) NKT-like cells in patients with T2DM and healthy controls (HC). In the quantification histograms, each dot represents a different individual and results are presented as the mean ± SEM; *P<0.05, ****P<0.0001, n.s., not significant.

To study the phenotype of NKT-like cells in T2DM, we analyzed by flow cytometry the expression of the major co-activating receptors, i.e. NKG2D, NKp46, and CD27, the early activation marker CD69, and the inhibitory receptors Tim-3, LAG-3, NKG2A, PD-1, and TIGIT, in circulating NKT-like cells from T2DM patients and HC. Results showed that the expression of NKG2D was significantly reduced in T2DM patients (**Figure 3A**), while no differences in NKp46 and CD69 expression levels were observed among groups (**Figure 3B, C**). The expression of CD27, a co-stimulatory immune-checkpoint receptor, also showed no significant variation in NKT-like cells from patients with T2DM compared to HC (**Figure 3D**). In turn, the expression of both Tim-3 and LAG-3 was significantly increased in NKT-like cells from T2DM patients (**Figure 4A, B**), while NKG2A, PD-1, and TIGIT levels were not affected, compared to HC (**Figure 4C-E**). Collectively, these results suggest that in T2DM patients circulating NKT-like cells are phenotypically exhausted, as shown by increased Tim-3 and LAG-3, and show also lower NKG2D expression.

### NKT-like cells are functionally defective in T2DM patients

**Figure 5:**
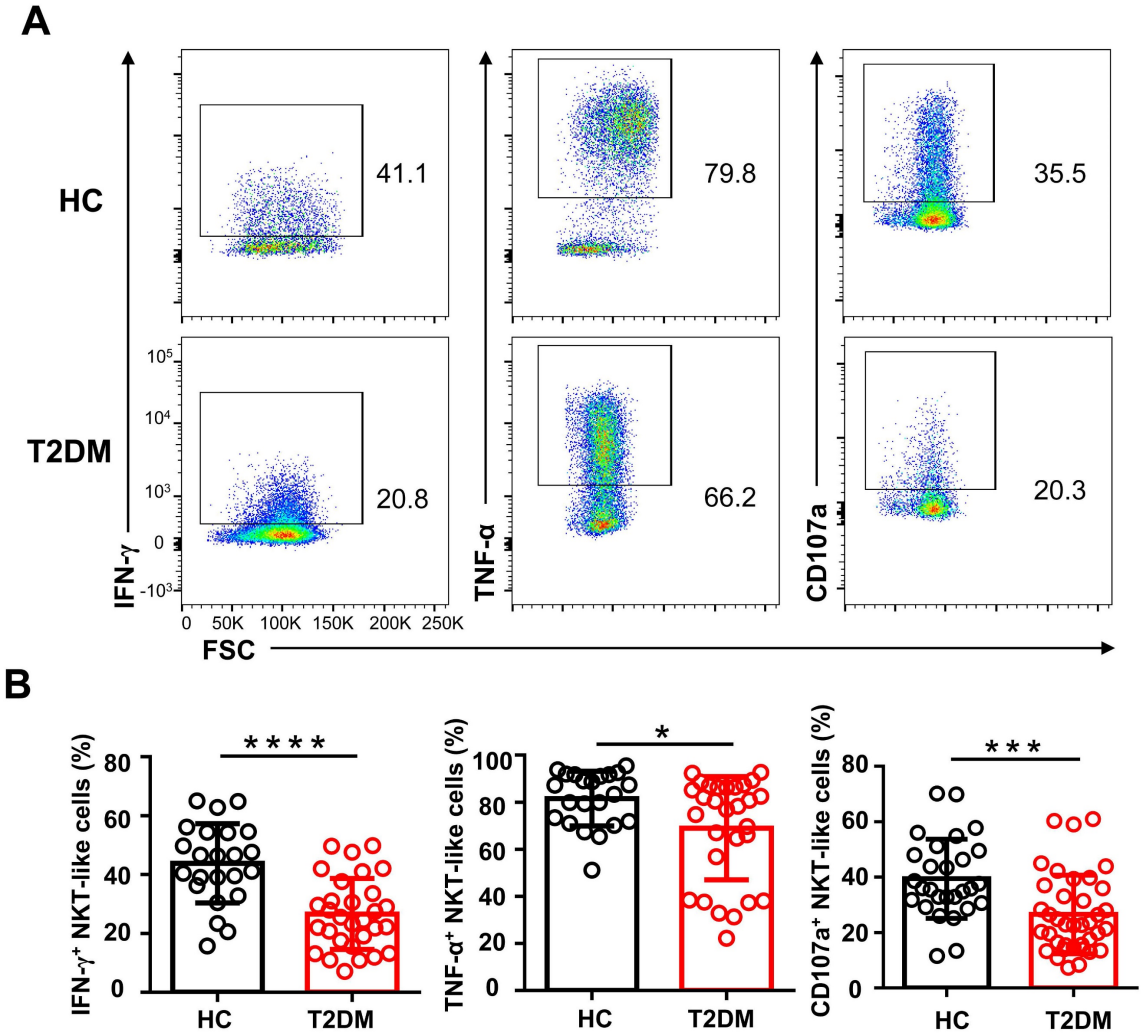
Circulating NKT-like cells from patients with T2DM show functional impairment. (**A**) PBMCs from T2DM and healthy controls (HC) were stimulated with PMA and ionomycin and the expression of IFN-γ, TNF-α, and CD107a by NKT-like cells was determined by flow cytometry. (**B**) Analysis of the percentage of IFN-γ^+^, TNF-α^+^, and CD107a^+^ NKT-like cells in patients with T2DM and HC. *P<0.05, ***P<0.001, ****P<0.0001.

To explore whether the phenotypic alterations observed in NKT-like cells from T2DM patients would lead to functional alterations, we examined by flow cytometry their cytokine production capacity upon PMA/ionomycin stimulation. As shown in **Figure 5A**, the percentage of IFN-γ^+^ and TNF-α^+^ NKT-like cells were significantly decreased in T2DM patients. Moreover, compared also to HC, NKT-like cells from T2DM patients exhibited impaired degranulation, as evidenced by decreased CD107a expression (**Figure 5A, B**). These data suggest that in patients with T2DM, NKT-like cells have a reduced capacity to produce cytokines and mediate cytotoxicity.

### Tim-3 upregulation leads to phenotypical alteration and functional exhaustion in NKT-like cells

**Figure 6:**
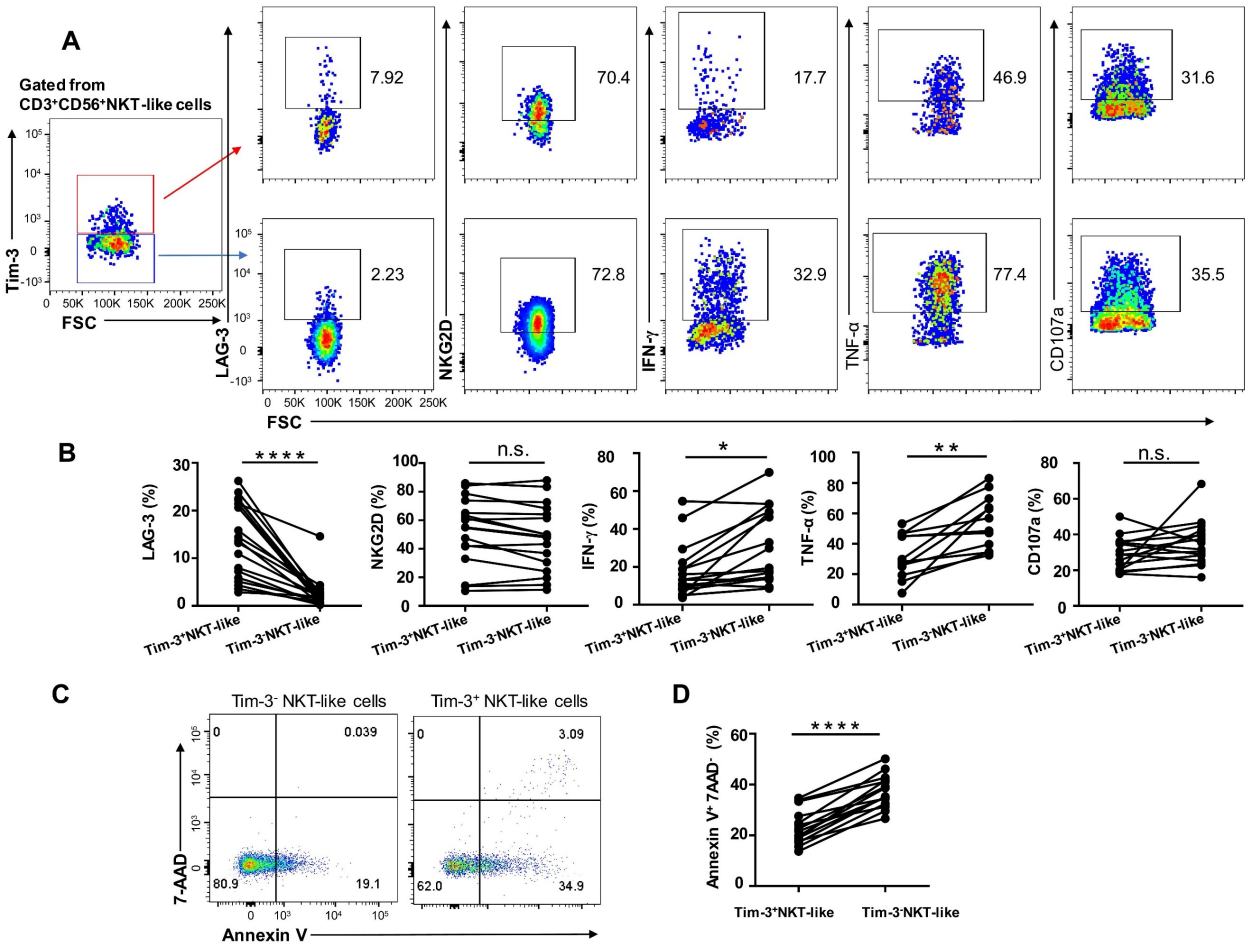
Tim-3^+^ NKT-like cells display weaker function than Tim-3^-^ NKT-like cells in patients with T2DM. (**A**) Representative flow cytometry plots of LAG-3, NKG2D, IFN-γ, TNF-α, and CD107a expression in Tim-3^+^ and Tim-3^-^ NKT-like cells from T2DM patients. (**B**) Proportions of LAG-3^+^, NKG2D^+^, IFN-γ^+^, TNF-a^+^, and CD107a^+^ Tim-3^+^ and Tim-3^-^ NKT-like cells from individual T2DM patients. (**C**) Representative flow cytometry plots of Annexin V^+^7-AAD^-^ staining in Tim-3^+^ and Tim-3- NKT-like cells from T2DM patients. (**D**) Proportions of Annexin V^+^ 7-AAD^-^ Tim-3^+^ and Tim-3^-^ NKT-like cells from individual T2DM patients. *P < 0.05. n.s., not significant.

Since expression levels of the inhibitory receptors Tim-3 and LAG-3 were upregulated in NKT-like cells from T2DM patients, we assessed the effect of Tim-3 expression on the functional and secretory capacities of such cells. We found that Tim-3^+^ NKT-like cells had a higher expression of LAG-3 compared with Tim-3^-^ NKT-like cells, while the expression of NKG2D was comparable (**Figure 6A, B**). In T2DM patients, Tim-3^+^ NKT-like cells exhibited also a much weaker capacity to produce TNF-α and IFN-γ, but similar capacity to produce CD107a, compared with Tim-3^-^ NKT-like cells (**Figure 6A, B**). Interestingly, further analysis showed that in T2DM patients Tim-3^+^ NKT-like cells showed a greater propensity to undergo apoptosis compared with Tim-3^-^ NKT-like cells (**Figure 6C, D**). Overall, these results suggest that in T2DM patients Tim-3^+^ NKT-like cells exhibit an exhausted phenotype, a suppressed effector function, and higher susceptibility to apoptosis.

### Tim-3 upregulation in NKT-like cells correlates with T2DM markers

**Figure 7:**
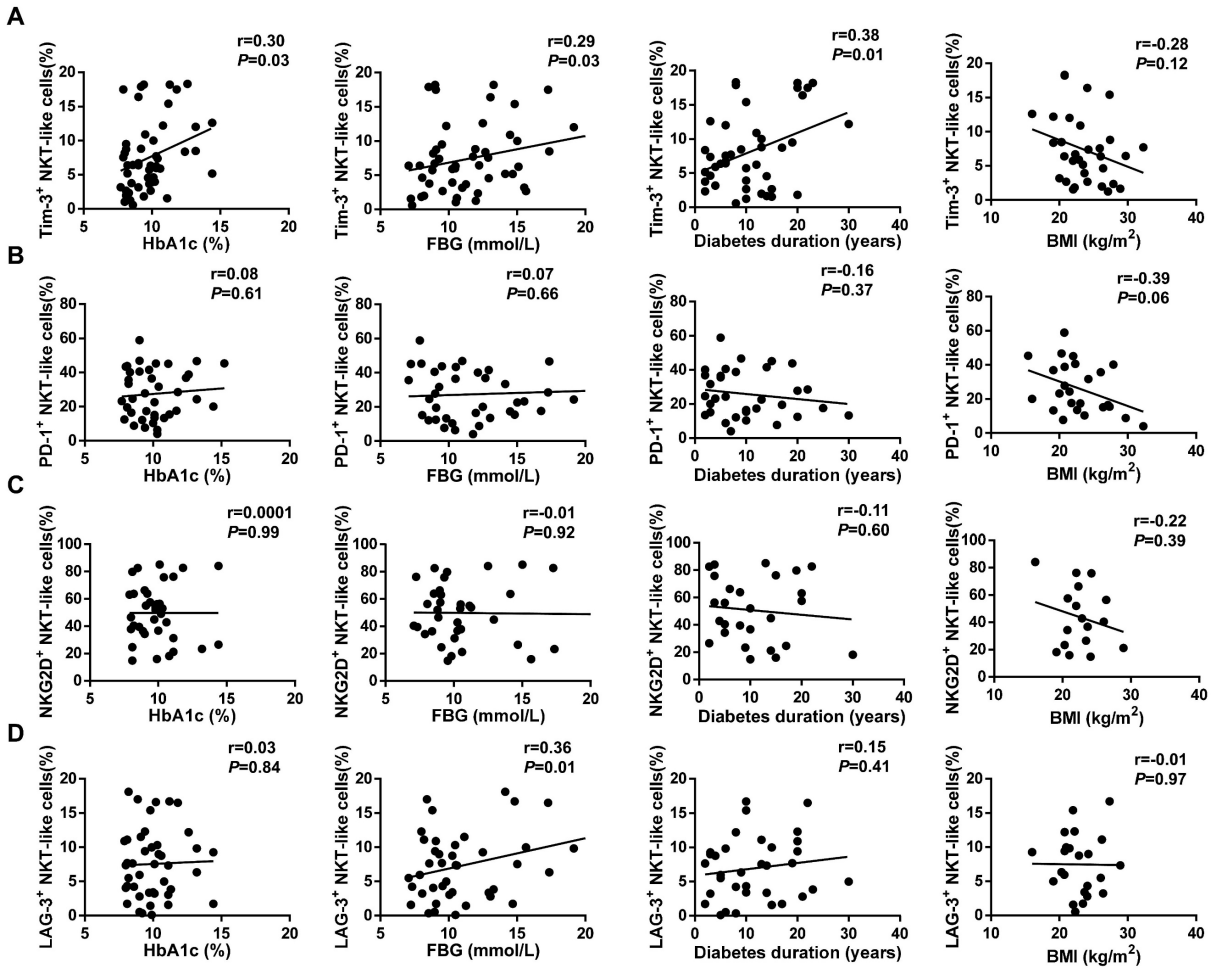
Correlation analysis of receptor expression on NKT-like cells and clinical indicators in T2DM patients. (**A**) Tim-3, (**B**) PD-1, (**C**) NKG2D, (**D**) LAG-3 expression on NKT-like cells and type 2 diabetes patients' glycated hemoglobin (HbA1c), fasting blood glucose (FBG), diabetes duration and body mass index (BMI). Correlation analysis were analyzed by Pearson analysis.

We next investigated the correlation between characteristic indicators of T2DM, namely glycated hemoglobin (HbA1c), fasting blood glucose (FBG), and body mass index (BMI), and receptor expression profiles in NKT-like cells. The expression of Tim-3 on circulating NKT-like cells from T2DM patients was positively correlated with both HbA1c (r=0.33, *P*=0.03) and FBG (r=0.29, *P*=0.03; **Figure 7A**). In contrast, there was no correlation between the expression of PD-1, NKG2D, or LAG-3 and either HbA1c or FBG levels (**Figure 7B-D**). To figure out whether high glucose responsible for the Tim-3 upregulation of NKT-like cells, we investigated the expression of Tim-3 on NKT-like cells in medium containing 5.5 mM, 25 mM, or 50 mM of glucose after incubation for 24h, 48h and 72h (**Supplementary Figure S2A-C**). We found that high glucose treatment did not directly affect Tim-3 expression on NKT-like cells. Importantly, a positive correlation was also detected between Tim-3 expression and the duration of diabetes (r=0.38, *P*=0.01; **Figure 7A**). In contrast, no correlation was found between the expression of other receptors and diabetes duration, nor between Tim-3, PD-1, NKG2D, or LAG-3 expression and BMI (**Figure 7A-D**). Moreover, we found that there was no correlation between diabetes duration and the expression of Tim-3 on CD56^+^NK cells or CD3^+^T cells (**Supplementary Figure S3A, B**). Overall, these results indicated that in poorly controlled T2DM overexpression of Tim-3 on circulating NKT-like may serve as a novel biological marker for diabetes duration.

## Discussion

T2DM patients are highly susceptible to infection and have an increased incidence of some tumors, possibly due to immune system dysfunction. Previous studies have shown that T2DM impairs the immune responses of neutrophils, NK cells, and T cells 18, 20. Long-term diabetes also increases the likelihood of many secondary injuries, and these complications are a significant cause of morbidity and mortality 21. Phenotypical alterations in CD3^+^CD56^+^ NKT-like cells have been correlated with susceptibility to various infections and tumors 9, 22, 23.

CD3^+^CD56^+^ NKT-like cells constitute a heterogeneous subpopulation of lymphocytes comprising TCRγδ^+^ T cells, MAIT cells, and a few invariant NKT (iNKT) cells, as well as CD4^+^ cells and CD8^+^ cells 4. NKT-like cells are important effectors responsible for controlling infections and combating tumor development. Since our understanding of the mechanisms underlying immune dysfunction in diabetes is still fragmentary, in this study we investigated the distribution and functional status of NKT-like cells in patients with T2DM. We detected a significantly lower frequency and absolute number of NKT-like cells in patients than in healthy controls. In pre-diabetes and new-onset T2DM patients, the frequency of circulating NKT-like cells has been reported as increased or normal compared with healthy controls 14, 24, 25. Differences in genetic background or diabetes duration among studied patients have been proposed to explain such dissimilar results. Our study also provides a plausible explanation for the observed decrease in NKT-like cell numbers in patients with T2DM, namely their greater tendency toward apoptosis without impaired proliferation. Indeed, previous studies have demonstrated that apoptosis of NK cells is enhanced in chronic diseases such as atherosclerosis and diabetes 18, 26.

In our study, CD8^+^cells were the largest subset of NKT-like cells, and their absolute number was significantly decreased in T2DM patients. Based on the typical frequency and co-receptor expression of MAIT, γδT cells and iNKT cells in human blood 27, 28, it is likely that these CD8^+^NKT-like cells mainly represent MAIT cells. In T2DM patients, circulating MAIT cell frequency was dramatically reduced, particularly in obese patients 29. The increased apoptosis of MAIT cells in mouse obesity model might explain the decreased MAIT cell frequency 30, which is consistent with the increased apoptosis of NKT-like cells observed in our study. CD56^+^CD3^+^NKT-like cells were largely composed of MAIT cells and γδT cells, whereas iNKT cells only make up about 1% 31. And there was no significant difference between T2DM patients and healthy controls in this study. Based the mouse model, iNKT cells in T1DM showed regulatory role 32, whereas the role of iNKT cells in T2DM was not clear.

Chronic low-grade inflammation is thought to be crucial for the development of insulin resistance and T2DM. In early phase of diabetes, the frequency of activated NKT-like cells (NKG2D^+^) was significantly higher than those in the healthy controls 14. Moreover, the counts of NKT-like cells producing granzyme and perforin was significantly increased in pre-diabetic patients compared to the T2DM group and healthy controls 24. In conclusion, activated NKT-like cells in new-onset T2DM patients and pre-diabetes, as one of the producers of chronic inflammation, contributed to the progression of diabetes. In our study, diabetes duration of our T2DM patients was relatively long (10.9 ± 7.1 years) in comparison to new-onset T2DM subjects. Our results suggested that NKT-like cells from T2DM patients showed an exhausted phenotype characterized by upregulation of Tim-3 and LAG-3, as well as downregulation of NKG2D. Co-inhibitory molecules such as PD-1, Tim-3, LAG-3, and TIGIT have been widely reported to inhibit T-cell and NK cell function 33-35. In addition, we observed functional impairment, denoted by decreased production of TNF-α, IFN-γ, and CD107a, in NKT-like cells from T2DM patients. Importantly, we observed that Tim-3^+^NKT-like cells was positively correlated to diabetes duration. Overall, NKT-like cells, in the microenvironment of chronic inflammation for ages, could up-regulate the expression of Tim-3 and eventually lead to exhaustion.

Tim-3 has been reported to promote immune tolerance through downregulation of Th1-dependent immune responses, whereas Tim-3 pathway blockade was shown to accelerate diabetes in NOD mice 36. In obese patients, Tim-3^+^CD8^+^ T cells were positively correlated with risk factors including BMI, body fat rate, and hipline 37. Meanwhile, Yan *et al.* showed that Tim-3 downregulation in CD14^+^ monocytes is negatively correlated with T2DM duration 38. In the present study, we demonstrated that the expression of Tim-3 on NKT-like cells from T2DM patients correlates positively with both HbA1c and FBG levels, two major markers of poor glycemic control, and also with disease duration. Since the latter might affect treatment effectiveness, it is important to find novel biological markers to aid its estimation.

In this investigation, high glucose treatment did not directly affect Tim-3 expression on NKT-like cells. The mechanisms regulating Tim-3 expression on NKT-like cells remain incompletely understood. The diabetes-related soluble factors, such as TNF-α, may play an important role in regulating Tim-3 expression 39. It has been reported that Tim-3 negatively regulates T cell responses by promoting T cell exhaustion via interaction with Galectin-9 and CD66a 40, 41. In our previous study, we revealed that there were no significant differences in Galectin-9 and CD66a/c/d/e expression in T cells, monocytes, dendritic cells, and NKT-like cells from T2DM patients compared to healthy volunteers 18. However, elevated serum Galectin-9 levels were observed in T2DM patients 18, 37, 42. Galectin-9 can be produced by diverse cell types, including lymphocytes, tumor cells, and endothelial cells. While the involvement of both immune and non-immune cells has been proposed, it is still not certain which cells are responsible for the elevated serum levels of soluble Galectin-9 in T2DM patients 43, 44.

## Conclusion

This study showed that in T2DM patients NKT-like cells display an exhausted phenotype and reduced functionality. Moreover, we report that the expression of Tim-3 on circulating NKT-like cells from T2DM patients correlates positively with diabetes duration. These finding suggest that Tim-3 might serve as a novel biomarker for disease duration in T2DM patients.

## Supplementary Material

Supplementary figures.

## Figures and Tables

**Table 1:** Clinical characteristics of enrolled T2DM patients and healthy controls.

Variables	T2DM patients N=54	Healthy controls N=33
Male	31(57.4%)	17(51.5%)
Age (years)	60.8±9.9	57.5±6.8
Diabetes duration (years)	10.9±7.1	-
BMI (kg/m^2^)	23.3±3.7	-
Fasting glycaemia(mmol/L)	12.4±4.0	5.3±0.3
HbA1c (%)	10.1±1.8	5.3±0.2
triglyceride (mmol/L)	1.9±1.1	1.4±0.9
total cholesterol (mmol/L)	4.4±1.1	4.7±0.9
HDL-cholesterol (mmol/L)	1.4±0.3	1.0±0.3
LDL-cholesterol (mmol/L)	2.6±0.9	2.7±0.8
Creatinine (umol/L)	68.6±30.4	65.9±12.9
Uric acid (umol/L)	311.5±95.7	341.8±80.9

Data were expressed as mean ± standard deviation (SD). Abbreviations: BMI: Body Mass Index; HbA1c: Glycosylated hemoglobin; HDL: high-density lipoprotein; LDL: low-density lipoprotein.

## Abbreviations

T2DM: Type 2 diabetes mellitus
HC: Healthy controls
PBMCs: Peripheral blood mononuclear cells
NKT cells: Nature killer T cells
NK cells: Nature killer cells
IFN-γ: Interferon-γ
TNF-α: Tumor necrosis factor α
PD-1: Programmed cell death protein 1
Tim-3: T-cell immunoglobulin and mucin structural domain protein 3
CTLA-4: Cytotoxic T-lymphocyte antigen 4
MHC: Major histocompatibility complex
LAG-3: Lymphocyte-activation gene 3
TIGIT: T cell immunoglobulin and ITIM domain
HbA1c: glycated hemoglobin
FBG: Fasting blood glucose
BMI: Body mass index
MAIT: mucosal-associated invariant T cells
FBS: Fetal bovine serum
iNKT: invariant NKT cells
NOD mice: Non-obese diabetic mice
α-GalCer: α-galactosylceramide
