# Supplementary material for: Dysfunction of circulating CD3+CD56+ NKT-like cells in type 2 diabetes mellitus
Source: Int J Med Sci. 2023 Apr 2;20(5):652–62. doi: 10.7150/ijms.83317 (PMC10110473; doi:10.7150/ijms.83317)
Supplement: Supplementary file 1 — Supplementary figures. [file ijmsv20p0652s1.pdf]

Supplementary Figures

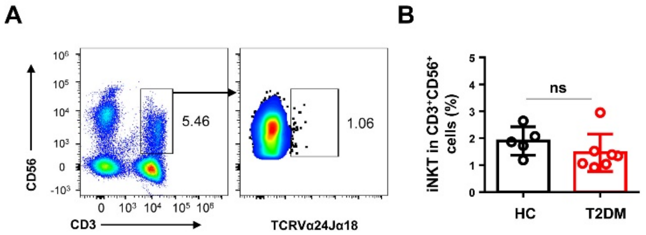

Supplementary Figure S1. Comparison of iNKT frequency in T2DM patients and HC. (**A**) Strategy for gating iNKT cells from NKT-like cells via flow cytometry. (**B**) Frequency of iNKT cells from patients with T2DM and healthy controls (HC). Each dot represents a different individual and results are presented as the mean ± SEM; n.s., not significant.

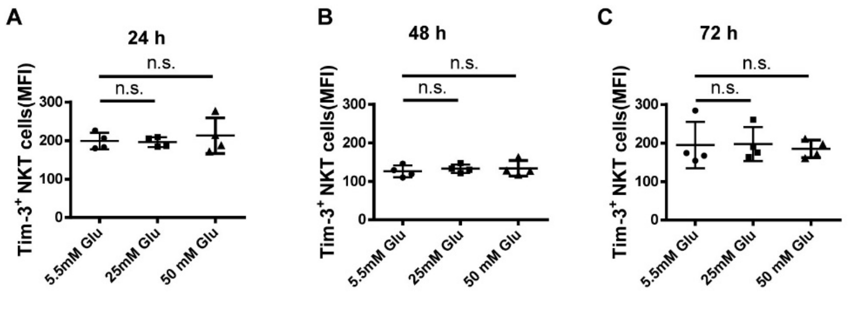

Supplementary Figure S2. High glucose treatment does not affect Tim-3 expression on NKT-like cells. PBMCs from healthy donors were cultured in RPMI-1640 medium containing 5.5 mM, 25 mM and 50 mM glucose. Expression of Tim-3 on NK cells after incubation 24h (**A**), 48h (**B**) and 72h (**C**). n.s., not significant.

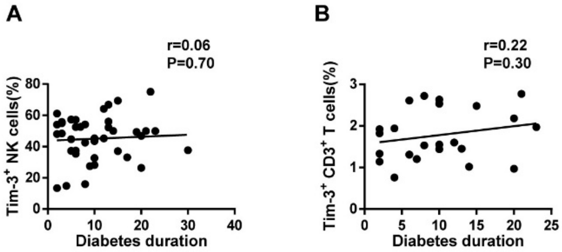

Supplementary Figure S3. Correlation analysis of diabetes duration and Tim-3 expression on NK cells (**A**) and T cells (**B**) in T2DM patients.
